# Supplementary material for: The Small GTPases in Fungal Signaling Conservation and Function
Source: Cells. 2021 Apr 28;10(5):1039. doi: 10.3390/cells10051039 (PMC8146680; doi:10.3390/cells10051039)
Supplement: Supplementary file 1 [file cells-10-01039-s001.zip › Material suplementario/Figure S3.pdf]

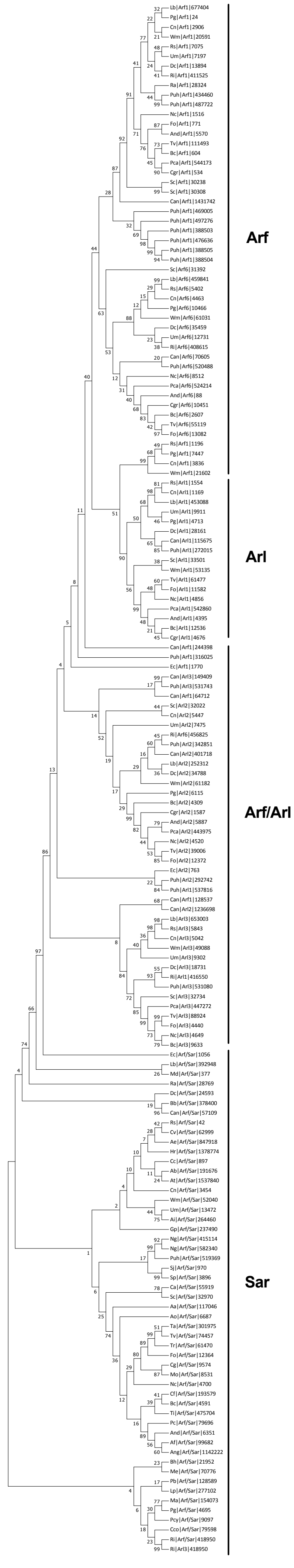

Lb|Arf1|677404
Pg|Arf1|24
Cn|Arf1|2906
Wm|Arf1|20591
Rs|Arf1|7075
Um|Arf1|7197
Dc|Arf1|13894
Ri|Arf1|411525
Ra|Arf1|28324
Puh|Arf1|434460
Puh|Arf1|487722
Nc|Arf1|1516
Fo|Arf1|771
And|Arf1|5570
Tv|Arf1|111493
Bc|Arf1|604
Pca|Arf1|544173
Cgr|Arf1|534
Sc|Arf1|30238
Sc|Arf1|30308
Can|Arf1|1431742
Puh|Arf1|469005
Puh|Arf1|497276
Puh|Arf1|388503
Puh|Arf1|476636
Puh|Arf1|388505
Puh|Arf1|388504
Sc|Arf6|31392
Lb|Arf6|459841
Rs|Arf6|5402
Cn|Arf6|4463
Pg|Arf6|10466
Wm|Arf6|61031
Dc|Arf6|35459
Um|Arf6|12731
Ri|Arf6|408615
Can|Arf6|70605
Puh|Arf6|520488
Nc|Arf6|8512
Pca|Arf6|524214
And|Arf6|88
Cgr|Arf6|10451
Bc|Arf6|2607
Tv|Arf6|55119
Fo|Arf6|13082
Rs|Arf1|1196
Pg|Arf1|7447
Cn|Arf1|3836
Wm|Arf1|21602
Arf
Rs|Arl1|1554
Cn|Arl1|1169
Lb|Arl1|453088
Um|Arl1|9911
Pg|Arl1|4713
Dc|Arl1|28161
Can|Arl1|115675
Puh|Arl1|272015
Sc|Arl1|33501
Wm|Arl1|53135
Tv|Arl1|61477
Fo|Arl1|11582
Nc|Arl1|4856
Pca|Arl1|542860
And|Arl1|4395
Bc|Arl1|12536
Cgr|Arl1|4676
Arl
Can|Arf1|244398
Puh|Arf1|316025
Ec|Arf1|1770
Can|Arl3|149409
Puh|Arl3|531743
Can|Arf1|64712
Sc|Arl2|32022
Cn|Arl2|5447
Um|Arl2|7475
Ri|Arf6|456825
Puh|Arl2|342851
Can|Arl2|401718
Lb|Arl2|252312
Dc|Arl2|34788
Wm|Arl2|61182
Pg|Arl2|6115
Bc|Arl2|4309
Cgr|Arl2|1587
And|Arl2|5887
Pca|Arl2|443975
Nc|Arl2|4520
Tv|Arl2|39006
Fo|Arl2|12372
Ec|Arl2|763
Puh|Arl2|292742
Puh|Arl1|537816
Can|Arf1|128537
Can|Arl2|1236698
Lb|Arl3|653003
Rs|Arl3|5843
Cn|Arl3|5042
Wm|Arl3|49088
Um|Arl3|9302
Dc|Arl3|18731
Ri|Arl1|416550
Puh|Arl3|531080
Sc|Arl3|32734
Pca|Arl3|447272
Tv|Arl3|88924
Fo|Arl3|4440
Nc|Arl3|4649
Bc|Arl3|9633
Arf/Arl
Ec|Arf/Sar|1056
Lb|Arf/Sar|392948
Md|Arf/Sar|377
Ra|Arf/Sar|28769
Dc|Arf/Sar|24593
Bb|Arf/Sar|378400
Can|Arf/Sar|57109
Rs|Arf/Sar|42
Cv|Arf/Sar|62999
Ae|Arf/Sar|847918
Hr|Arf/Sar|1378774
Cc|Arf/Sar|897
Ab|Arf/Sar|191676
At|Arf/Sar|1537840
Cn|Arf/Sar|3454
Wm|Arf/Sar|52040
Um|Arf/Sar|13472
Ai|Arf/Sar|264460
Gp|Arf/Sar|237490
Ng|Arf/Sar|415114
Ng|Arf/Sar|582340
Puh|Arf/Sar|519369
Sj|Arf/Sar|970
Sp|Arf/Sar|3896
Ca|Arf/Sar|55919
Sc|Arf/Sar|32970
Aa|Arf/Sar|117046
Ao|Arf/Sar|6687
Ta|Arf/Sar|301975
Tv|Arf/Sar|74457
Tr|Arf/Sar|61470
Fo|Arf/Sar|12364
Cg|Arf/Sar|9574
Mo|Arf/Sar|8531
Nc|Arf/Sar|4700
Cf|Arf/Sar|193579
Bc|Arf/Sar|4591
Ti|Arf/Sar|475704
Pc|Arf/Sar|79696
And|Arf/Sar|6351
Af|Arf/Sar|99682
Ang|Arf/Sar|1142222
Bh|Arf/Sar|21952
Me|Arf/Sar|70776
Pb|Arf/Sar|128589
Lp|Arf/Sar|277102
Ma|Arf/Sar|154073
Pg|Arf/Sar|4695
Pcy|Arf/Sar|9097
Cco|Arf/Sar|79598
Ri|Arf/Sar|418950
Ri|Arl3|418950
Sar
