# Supplementary figures and images for: The Small GTPases in Fungal Signaling Conservation and Function
Source: Cells. 2021 Apr 28;10(5):1039. doi: 10.3390/cells10051039 (PMC8146680; doi:10.3390/cells10051039)

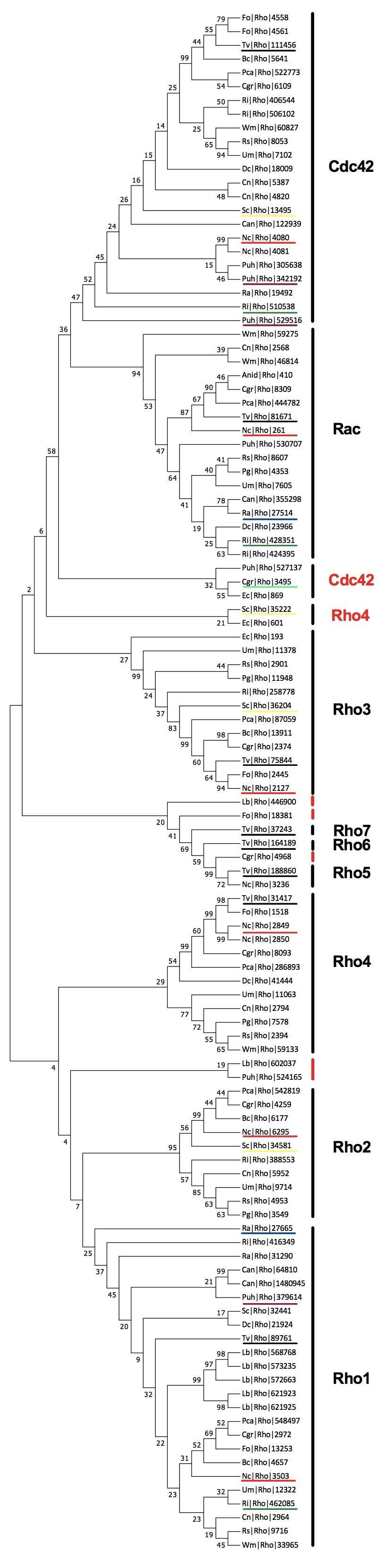

Supplement: Supplementary file 1 [file cells-10-01039-s001.zip › Material suplementario/Figure S1.tiff]

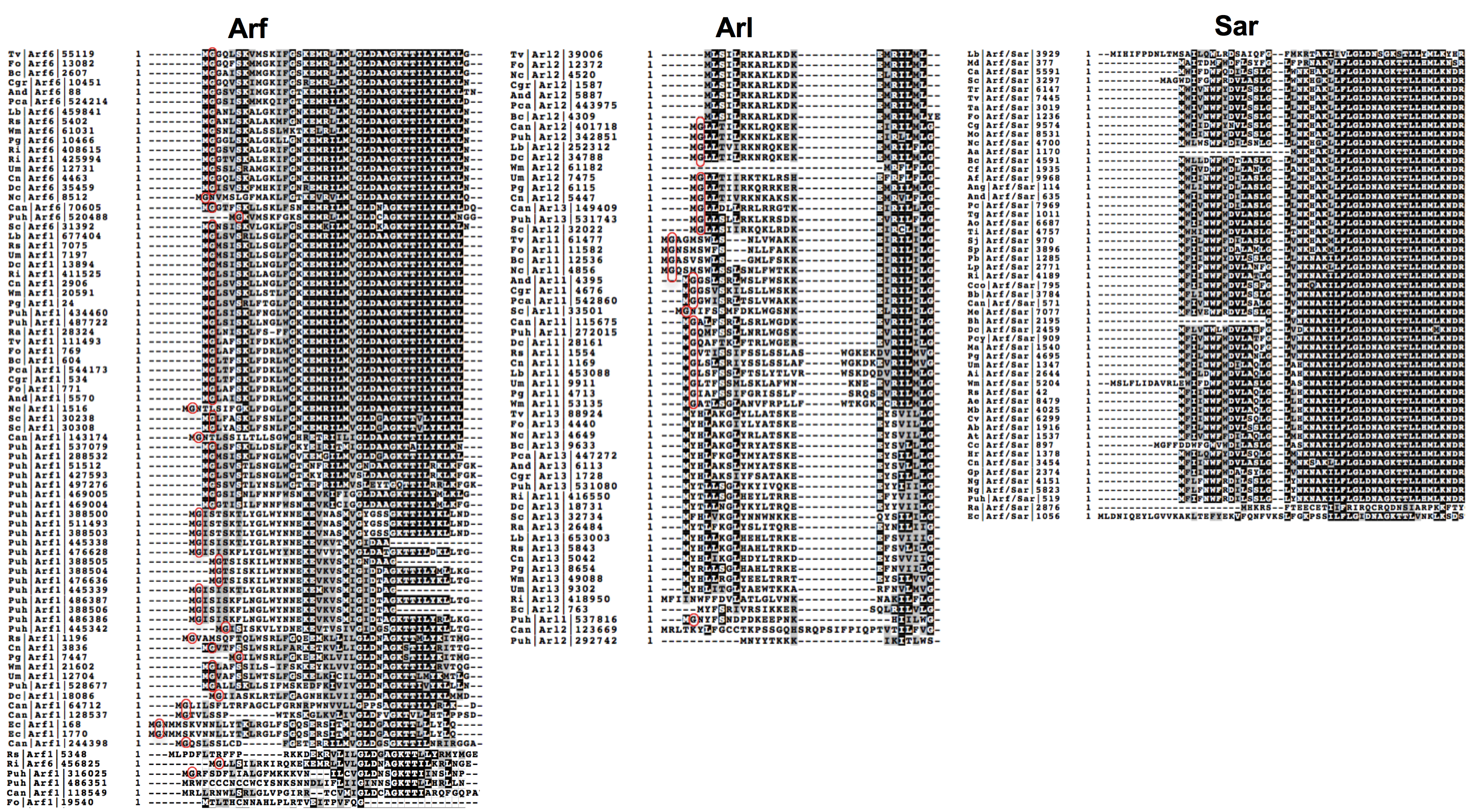

Supplement: Supplementary file 1 [file cells-10-01039-s001.zip › Material suplementario/Figure S2.tiff]

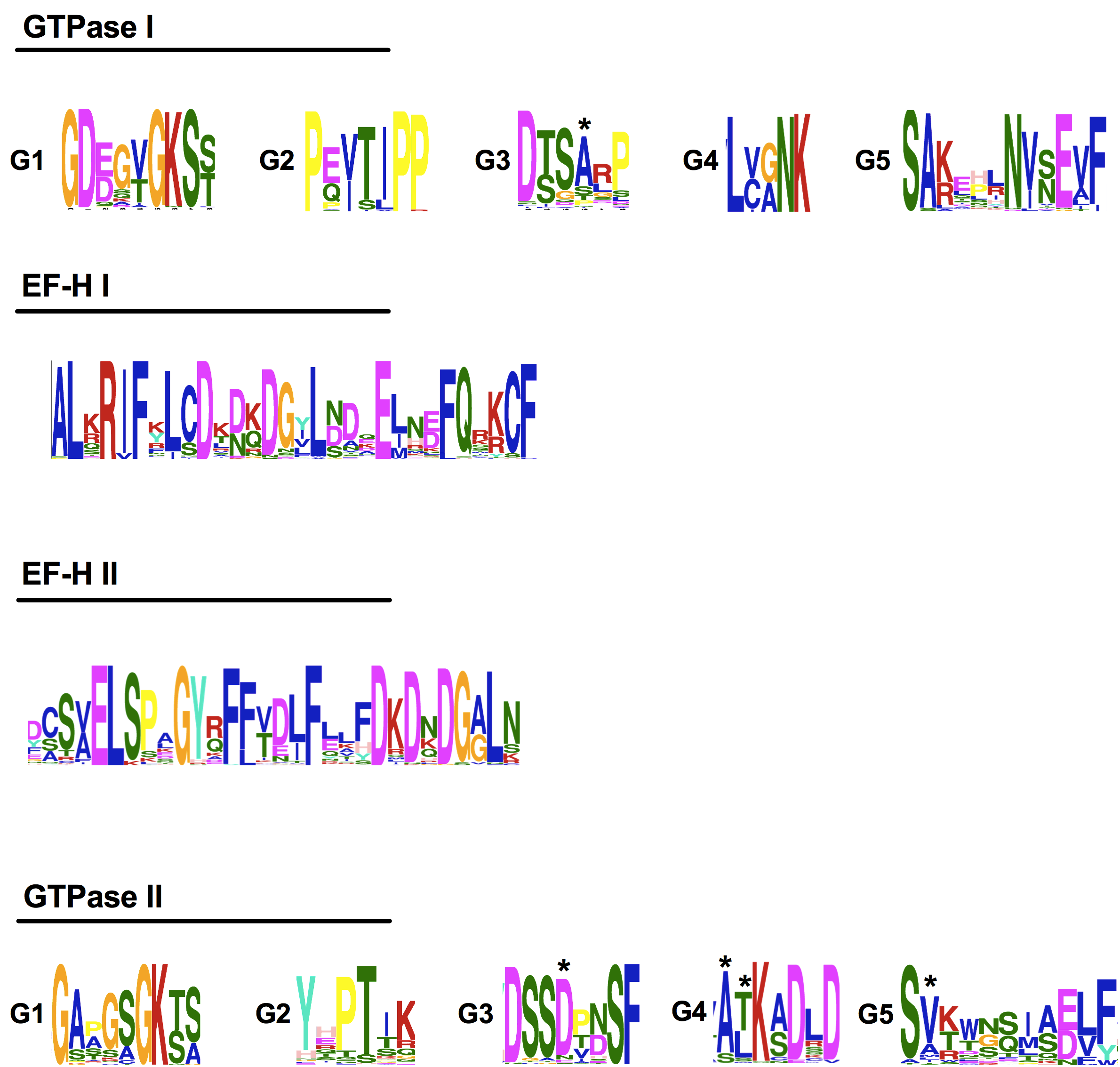

Supplement: Supplementary file 1 [file cells-10-01039-s001.zip › Material suplementario/Figure S4.tiff]
